# Heat-Shock Proteins in Neuroinflammation

**DOI:** 10.3389/fphar.2019.00920

**Published:** 2019-08-27

**Authors:** Brigitta Dukay, Bálint Csoboz, Melinda E. Tóth

**Affiliations:** ^1^Institute of Biochemistry, Biological Research Centre, Hungarian Academy of Sciences, Szeged, Hungary; ^2^Doctoral School in Biology, Faculty of Science and Informatics, University of Szeged, Szeged, Hungary

**Keywords:** neuroinflammation, heat shock proteins, heat shock response, diseases of the central nervous system, extracellular heat shock proteins, inflammation modulation

## Abstract

The heat-shock response, one of the main pro-survival mechanisms of a living organism, has evolved as the biochemical response of cells to cope with heat stress. The most well-characterized aspect of the heat-shock response is the accumulation of a conserved set of proteins termed heat-shock proteins (HSPs). HSPs are key players in protein homeostasis acting as chaperones by aiding the folding and assembly of nascent proteins and protecting against protein aggregation. HSPs have been associated with neurological diseases in the context of their chaperone activity, as they were found to suppress the aggregation of misfolded toxic proteins. In recent times, HSPs have proven to have functions apart from the classical molecular chaperoning in that they play a role in a wider scale of neurological disorders by modulating neuronal survival, inflammation, and disease-specific signaling processes. HSPs are gaining importance based on their ability to fine-tune inflammation and act as immune modulators in various bodily fluids. However, their effect on neuroinflammation processes is not yet fully understood. In this review, we summarize the role of neuroinflammation in acute and chronic pathological conditions affecting the brain. Moreover, we seek to explore the existing literature on HSP-mediated inflammatory function within the central nervous system and compare the function of these proteins when they are localized intracellularly compared to being present in the extracellular milieu.

## Introduction

Neuroinflammation is a well-known feature of different acute brain disorders including ischemic stroke, traumatic brain injury, and chronic neurodegenerative diseases such as Alzheimer’s (AD) and Parkinson’s (PD) diseases (Kim and Yenari, 2013; Banjara and Ghosh, 2017). It is characterized by an increased level of cytokines and chemokines with concomitant activation of glial cells and the infiltration of leukocytes (Kim and Yenari, 2013).

Activation of the inflammatory cascades within these cells is mediated by specialized pattern-recognition receptors (PRRs) like toll-like receptors (TLRs) and NOD-like receptors (Banjara and Ghosh, 2017). PRRs recognize different danger signals triggering immune response. These danger signals can be external, mainly pathogen-derived molecules (pathogen-associated molecular patterns-PAMPs). On the other hand, tissue injury after ischemic stroke or during chronic neurodegenerative diseases can lead to sterile inflammation. In these conditions, the release of endogenous damage–associated molecules (danger-associated molecular pattern-DAMP) activates the inflammatory cascade (Banjara and Ghosh, 2017). DAMPs could flow out from necrotic cells or they can be secreted specifically by damaged cells to trigger the immune response to clean cell debris and initiate tissue repair (Giuliano et al., 2011).

DAMPs are very diverse regarding their origin and chemical properties. They include uric acid, extracellular ATP, mitochondrial DNA, misfolded proteins like β-amyloid (Aβ), glycoproteins, hormones, and extracellular RNA (Patel, 2018). Moreover, heat-shock proteins (HSPs) appear to be ideal DAMPs, as they are highly conserved molecules, one of the most abundant intracellular proteins whose expression is further increased upon tissue injury like thermal or oxidative stress or in response to infections. As molecular chaperones, HSPs are able to bind several peptides suggesting that they can be involved in antigen presentation. Moreover, HSPs themselves are able to trigger the inflammatory response (Giuliano et al., 2011).

DAMPs can bind to the PRRs of microglia and other immune cells leading to the stimulation of inflammatory cascades by the activation of different pro-inflammatory transcription factors like nuclear factor kappa B (NF-κB) (Kim and Yenari, 2013; Banjara and Ghosh, 2017). Under normal conditions, the inactive heterodimeric NF-κB is bound by its inhibitor NF-κB inhibitor beta (IkB) in the cytosol. Activation of the inflammatory cascade results in the phosphorylation and degradation of IkB, releasing NF-κB. The activated transcription factor then moves to the nucleus, initiating the expression of several inflammatory factors like cytokines and chemokines (Kim and Yenari, 2013).

The condition of the brain tissue is constantly monitored by glial cells, primarily microglia and astrocytes (Banjara and Ghosh, 2017). Brain-resident microglia cells are the first responders to central nervous system (CNS) damage; they accumulate within a short period of time in the injured area and begin the phagocytic removal of myelin debris, which is important because this debris can release axonal regrowth–inhibiting factors. Moreover, activated microglia also play an important role in glial scar formation and in support of the blood–brain barrier (BBB) (Mosley and Cuzner, 1996; Schwab, 2004; Davalos et al., 2005; Lou et al., 2016; Xu et al., 2019). However, through their TLRs, DAMPs activate the pro-inflammatory phenotype of microglia besides the early phagocytic phenotype, which is destructive to the brain tissue since it can produce pro-inflammatory cytokines, chemokines, complement system components, free radicals, and matrix metalloproteases (MMPs) which could result in an increase in BBB permeability and apoptosis (Walker et al., 1995; Jack et al., 2005; Yenari et al., 2006; del Zoppo et al., 2012; Kawabori and Yenari, 2015). Activation of microglia is the first step in the inflammatory response of the brain, followed by the activation and proliferation of astrocytes, contributing to an even more robust secretion of pro-inflammatory signaling molecules (Banjara and Ghosh, 2017). The released chemokines play a major role in the activation of peripheral immune cells (Glabinski et al., 1995). In response to inflammatory factors, neutrophils rapidly infiltrate into the affected brain areas. Like glia, they produce inflammatory mediators and reactive oxygen species and can be the source of high MMP levels in the injured area, thereby enhancing neurotoxicity, contributing to vascular permeability, and causing cerebral edema. On the other hand, by phagocytosis and production of growth factors, neutrophils can also take part in restoration of the damaged area (Schoettle et al., 1990; Soares et al., 1995; Nguyen et al., 2007; Rosell et al., 2008; Liu et al., 2018a). Peripheral monocytes are also able to enter the brain parenchyma a few hours after injury. When the peripheral monocyte encounters traumatized or infected tissue, it is differentiated into a macrophage or a dendritic cell. The infiltrating macrophages are primarily cytotoxic, but their function can vary depending on whether the pro- or the anti-inflammatory phenotype is dominant; therefore, they can have a role in phagocytosis, cytokine/chemokine release, antigen presentation, astroglial scar formation, and tissue repair (Girard et al., 2013; Vogel et al., 2014; Gliem et al., 2016; Frik et al., 2018; Malo et al., 2018). Dendritic cells are important in both innate and adaptive immune responses, but their predominant role is antigen presenting by which antigen-specific T cells are being activated and migrated into the brain (Felger et al., 2010; Malo et al., 2018). Thus, the switch from non-specific innate immune response to the adaptive response is triggered by the appearance of T cells, which are activated *via* antigens presented by dendritic cells, macrophages, and microglia. Different types of T lymphocytes have different roles. CD4 + (“helper”) T cells control the adaptive immune response, while CD8 + (“killer”) T cells kill infected and damaged cells. T-cell infiltration after injury is mostly neurotoxic: it releases interferon gamma (INFγ) and other inflammatory cytokines, but it can also have a protective role. T regulatory cells can protect against inflammation in many ways, including expression of tumor growth factor beta (TGF-β) and interleukin-10 (IL-10) (Ford et al., 1996; Liesz et al., 2009; Gill and Veltkamp, 2016; Malo et al., 2018).

### Role of Neuroinflammation in Different Acute and Chronic CNS Diseases

Inflammation has a key role in most acute and neurodegenerative CNS disorders. However, it is complicated to define if inflammation is a cause or consequence of neuronal cell death in different CNS diseases. Neuroinflammation can naturally occur in response to many acute damaging effects, such as pathogenic invasion or tissue injury being the first line of defense of the brain tissue (Swaroop et al., 2016; Banjara and Ghosh, 2017). Activated immune cells combat against pathogens and eliminate damaged tissue, thereby, preventing the spread of infection and necrosis. However, we should not neglect the Janus-faced nature of inflammation. In addition to protective functions, an excessive, over-activated inflammatory response may lead to further neuronal damage (reviewed recently in Le et al., 2016; Banjara and Ghosh, 2017; Sochocka et al., 2017). In the case of chronic, age-related neurodegenerative diseases, inflammation seems to have a central role in the pathology of the disease. Since the development of these disorders is a slow process, the underlying pathophysiological mechanisms induce a constant immune response in the brain, leading to chronic inflammation. While a rapid, acute course of inflammation which induces an immune reaction within a relatively short period of time can have beneficial effects, prolonged inflammation triggered by continuously present insults usually leads to the destruction of brain tissue. Additionally, persistent activation interferes with the normal, beneficial housekeeping functions of resident cells of the brain, such as microglia and astrocytes. Although these cells can be involved in the elimination of pathological protein aggregates, they go through cellular senescence with age and are less able to perform their physiological functions (Floden and Combs, 2011; Caldeira et al., 2017; Gomez-Arboledas et al., 2018). Along with aging, the integrity of the vascular network also decreases (Elahy et al., 2015; Montagne et al., 2015), allowing systemic immune processes to affect the brain, which is in turn no longer immune-privileged. These processes could lead to the dysregulation of immune mechanisms in the CNS and to the development of a pro-inflammatory milieu, which could advance the progression of neurological diseases and neurodegeneration (reviewed in Costantini et al., 2018).

The tissue injury caused by acute, sterile brain-damaging conditions like ischemic stroke and by traumatic brain injury could be very similar to each other. After cerebral artery occlusion, a core region is formed where the blood flow ceases and the subsequent glucose and oxygen deficiency completely eliminate the neurons and glial cells. This is surrounded by a so-called penumbra, where there is still a limited blood perfusion which already damages the cells but does not lead to cell death (Zhang et al., 1997; Lee et al., 2000). Similarly, in the traumatized brain area, hypoperfusion and local ischemia also develop. In both cases, the impacts are followed by reperfusion, which is indispensable in the restoration of brain oxygen and nutrient supply, yet causes inflammatory processes and secondary tissue damage (Coles, 2004; Lin et al., 2016). After injury, the amount of the two major pro-inflammatory cytokines, IL-1β and tumor necrosis factor (TNF)-α, increases in the core and penumbra regions after a few hours by expression of resident CNS cells. This activates additional microglia and astrocyte cells, increases the production of chemokines, the level of apoptosis, the permeability of the BBB, and edema formation (Feuerstein et al., 1994; Sairanen et al., 2001; Rothwell, 2003; Hosomi et al., 2005; Lu et al., 2005; Sozen et al., 2009). Under experimental conditions of stroke and traumatic brain injury, the suppression of pro-inflammatory cytokines could prevent the loss of interneurons and oligodendrocytes, reduce the infarct area, thereby alleviating cognitive impairment, and also affect the efficacy of repair mechanisms in the long term (Scherbel et al., 1999; Flygt et al., 2018; Newell et al., 2018; Wang et al., 2018a; Wong et al., 2019). In addition to the therapeutic inhibition of pro-inflammatory cytokines, the targeting of anti-inflammatory cytokines could also be potentially relevant in the therapy of neurological diseases. In animal experiments, IL-10 administration decreased the size of the infarct area after middle cerebral artery occlusion and down-regulated pro-inflammatory signaling, while following traumatic brain injury, it also reduced TNF-α and IL-1 levels and increased neural repair processes (Knoblach and Faden, 1998; Spera et al., 1998; Liesz et al., 2014).

**Figure 1:**
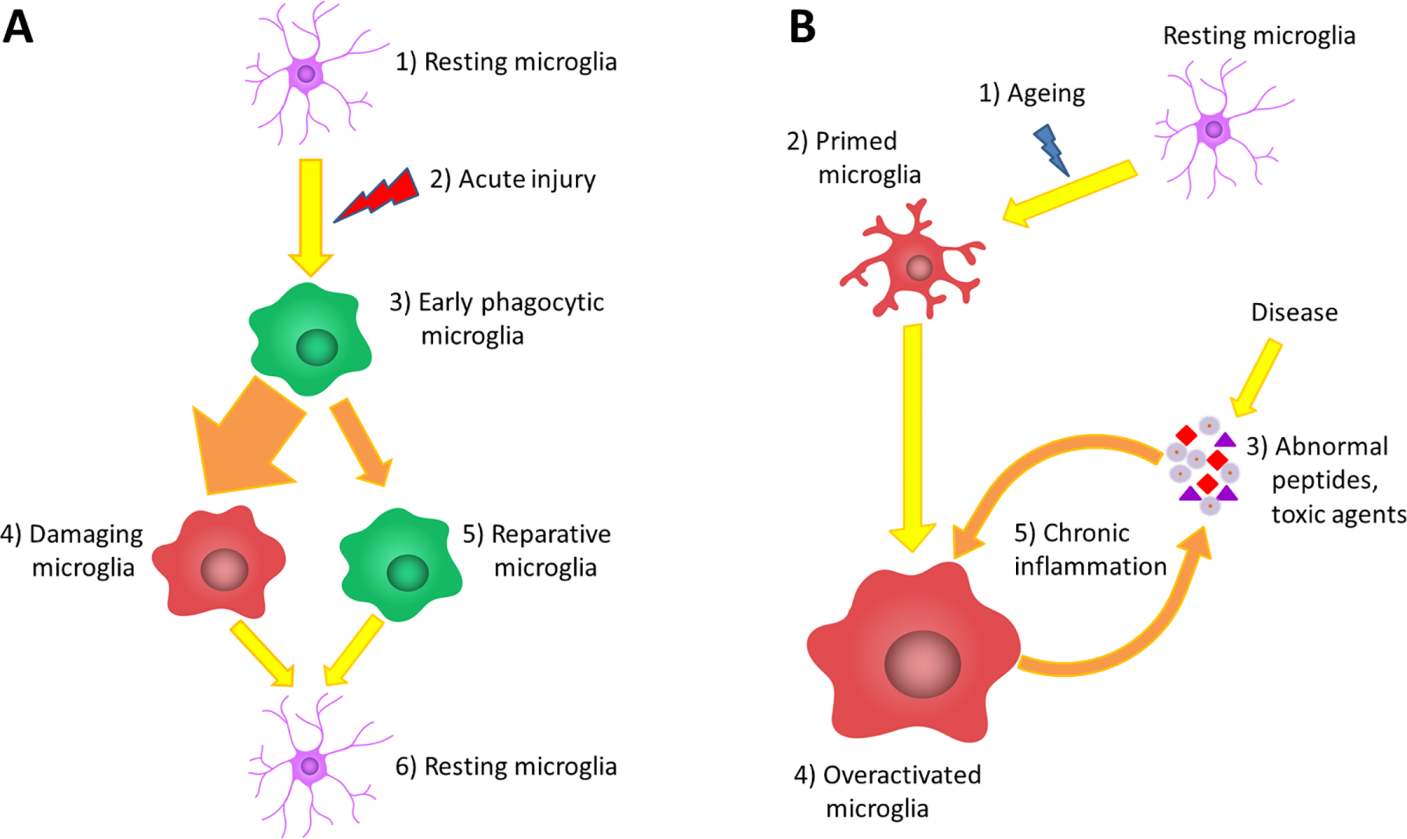
The role of microglia in acute and chronic inflammations. **(A)** Under normal circumstances, the brain tissue is constantly monitored by resting microglia cells (1). Acute brain damage triggers dynamic microglia activation. This is a linear process that will decline over time (2). First, an early phagocytic phenotype accumulates in the injured area and begins to remove the debris (3). However, the majority of microglia rapidly converts to a pro-inflammatory phenotype *via* classical activation, which is neurotoxic, induces apoptosis and exacerbates inflammatory processes (4). At the same time, some microglia get alternatively activated and exert a neuroprotective effect to counteract the pro-inflammatory processes (5). After the resolution of the inflammatory cascades, microglia return to resting state and continue the tissue surveillance (6). **(B)** Due to physiological aging, the basic activation state of microglia is increased (2), which is associated with elevated production of cytokines and inflammatory mediators. These so-called primed microglia cells show an elevated responsiveness (3) and potentially can be more stimulated by binding disease-associated proteins and other toxic agents (4). The over-activation results in the impairment of certain microglial functions lowering their ability to eliminate the toxic protein aggregates; however, in line with their phagocytic dysfunction, their productivity of inflammatory cytokines further increases (5). This negative correlation between the expression of pro-inflammatory cytokines and the clearance of abnormal proteins generates the vicious cycle of chronic inflammation, which is a characteristic of neurodegenerative diseases (6).

Recently, a potential neuroprotective role for microglial cells was described when the inhibition of microglia has led to increased astrocyte activation and increased infarct size by 60% after focal brain injury (Szalay et al., 2016; Jin et al., 2017). The role of microglia in brain injury could be dual, as they are able to form a barrier between the injured and healthy tissue with their projections and support the glia limitans (Davalos et al., 2005; Roth et al., 2014), while activated microglial cells have been shown to be neurotoxic and to induce apoptosis and exacerbate inflammatory processes in other studies (Kaushal and Schlichter, 2008; Hu et al., 2012; **Figure 1A**).

Acute trauma reduces the integrity of the cerebral microvascular network (Chodobski et al., 2011). In addition, the released chemokines facilitate the entry of neutrophils, monocytes, and T cells (Dimitrijevic et al., 2007; Llovera et al., 2017). Experimental data from numerous studies have confirmed that the influx of neutrophils and monocytes is predominantly pro-inflammatory and harmful. For example, after intracerebral hemorrhage formation, neutrophils were shown to contribute to MMP-9-mediated BBB damage, to white matter destruction, and to the increased damaging effect of glial cells to the brain (Moxon-Emre and Schlichter, 2011). However, it is important that peripheral macrophages with high phagocytic capacity also appear 72 h after middle cerebral artery occlusion (Ritzel et al., 2015). Their beneficial role is demonstrated by the fact that, after ischemia, the use of the chemokine receptor type 2 (CCR2) antibody reduced the level of anti-inflammatory cytokines and long-term repair processes (Wattananit et al., 2016). The appearance of T cells in the acute phase exacerbates brain damage; accordingly, T cells are crucial to the development of secondary neurodegeneration. This is supported by experiments, in which the inhibition of IL-17 produced by γδ T-cells, or IL-21 expressed by CD4+ T-cells successfully reduced the infarct size and protected against middle cerebral artery occlusion (Clarkson et al., 2014; Arunachalam et al., 2017). On the other hand, regulatory T cells activated in later phase were proved to be protective. In an animal model of stroke, regulatory T cells reduced the level of astrocyte activation and increased the production of anti-inflammatory cytokines (Ito et al., 2019).

In many age-related neurodegenerative disorders, aggregation-prone proteins accumulate in neurons, such as Aβ and tau in AD, α-synuclein in PD or huntingtin in Huntington’s disease (HD). These proteins tend to form insoluble inclusion bodies within the brain tissue, which can initiate a chronic inflammatory response (Khandelwal et al., 2011; Yang et al., 2017). The importance of the role of inflammation and microglia function in the pathogenesis of neurodegenerative diseases is supported by large-scale genetic studies (Malik et al., 2015; Gelders et al., 2018). Several single nucleotide polymorphisms have been identified associated with AD in genes related to microglial phagocytosis or the complement system, such as ABCA7, CD33, and CR1 (Lambert et al., 2009; Hollingworth et al., 2011; Naj et al., 2011). A missense mutation in the gene expressing triggering receptor expressed on myeloid cells 2 (TREM2) was found to be a significant risk factor not only for AD (Jonsson et al., 2013) but for frontotemporal dementia and PD as well (Rayaprolu et al., 2013). TREM2 is involved in the activation of microglial phagocytosis without the increase of cytokine production; therefore, it can possibly stimulate the elimination of Aβ plaques and apoptotic neurons (Rayaprolu et al., 2013; Savage et al., 2015). In the brain of AD patients, microglia were proved to migrate toward the amyloid plaques and to become more stimulated by binding to Aβ. This sustained microglia activation results in the impairment of microglial functions, like motility and phagocytosis, lowering their ability to eliminate these toxic protein aggregates from the brain (Su et al., 2008; Stewart et al., 2010; Krabbe et al., 2013). In line with the loss of their phagocytic capacity by decreased scavenger receptors and amyloid-degrading enzymes, the productivity of inflammatory cytokines further increased in an aged APP/PS1 animal model of AD. This negative correlation between the expressions of IL-1β, TNF-α, and Aβ clearance generates neuronal damage (Hickman et al., 2008; **Figure 1B**). Earlier results showed that IL-1β expression can further stimulate the synthesis and cleavage of amyloid precursor protein, leading to Aβ deposition, which activates the vicious cycle of Aβ-induced inflammation (Goldgaber et al., 1989; Buxbaum et al., 1992). Although in the 3xTg-AD model, microglial activation by IL-1β was shown to be useful in removing amyloid plaques; at the same time, it also increased the neurotoxicity of the tau protein (Ghosh et al., 2013). Extracellularly secreted hyperphosphorylated tau can also trigger the activation of microglia and astrocytes with a concomitant increase of cytokine release (reviewed in Laurent et al., 2018). In turn, the increased inflammation and glia activation were shown to provoke tau hyperphosphorylation exacerbating tau neuropathology in 3xTg-AD mice (Kitazawa et al., 2005). Activated microglial cells with decreased ramification have been detected in the brain of HD patients and in the YAC128 mouse model of the disease as well (Politis et al., 2011; Franciosi et al., 2012). *In vitro* and transgenic mouse studies showed that healthy microglia can help to remove dysfunctional neuronal cells and restrain the progression of the disease (Kraft et al., 2012; Benraiss et al., 2016). In contrast, transplantation of mutant huntingtin-expressing human glial cells into normal mice worsened their motoric performance (Benraiss et al., 2016). These results suggest that, similarly to AD and PD, abnormal microglial function has a crucial role in the pathogenesis of HD.

In addition, the inflammasomes of infiltrating monocytes and in microglia can be activated by Aβ, which further enhances the production of IL-1β and IL-18 (Halle et al., 2008; Saresella et al., 2016). TNF-α was also shown to be elevated in the cerebrospinal fluid of AD patients and was described to be a risk factor in PD (Tarkowski et al., 2003; Lindenau et al., 2017). In the APP23 transgenic mouse model of AD, suppression of the TNF-α receptor 1 diminished Aβ production and plaque formation by reducing BACE1 activity, which, in parallel, reduced disease-associated cognitive dysfunctions (He et al., 2007). The role of the anti-inflammatory IL-10 is controversial, as its elevated expression lowers the level of pro-inflammatory cytokines and chemokines, which is important for restoring immune balance, yet in excess, it restrains the activated microglia around plaques causing reduced Aβ phagocytosis (Szczepanik et al., 2001; Guillot-Sestier et al., 2015).

Beside microglia, astrocytes can also contribute to the neurotoxicity triggered by disease-associated proteins. In the brain of AD and PD patients, reactive astrogliosis appears, and its distribution is associated with the presence of α-synuclein containing Lewis bodies and Aβ senile plaques (Braak et al., 2007; Medeiros and LaFerla, 2013). Since astrocytes take part in the formation of the BBB, their activation in old mice compared to young ones promoted barrier dysfunction, which occurs during normal ageing, increasing the risk of disease development (Elahy et al., 2015). Primary astrocytes isolated from AD mice showed a highly induced BACE-1 expression and amyloid precursor protein secretion upon IFNγ treatment, presuming the role of astrocytes in amyloidosis (Hong et al., 2003). In contrast, a study in which wild-type astrocytes were transplanted into the brain of a mouse with AD demonstrates the capacity of astrocytes to degrade Aβ (Pihlaja et al., 2011). In PDGF α-synuclein transgenic mice, astrocytes took up neuronal cell-derived α-synuclein which stimulated the release of pro-inflammatory cytokines, chemokines, and other inflammatory mediators (Lee et al., 2010).

Peripheral immune cells, like macrophages, were shown to assemble around Aβ deposits to eliminate them in the mouse model of AD. Furthermore, in different models of AD, an increased load of amyloid occurred after the restriction of mononuclear phagocyte infiltration into the brain or with the removal of CD11b+ macrophages from the parenchyma (Simard et al., 2006; El Khoury et al., 2007). In contrast, macrophages from AD patients presented a limited internalization of Aβ, suggesting a phagocytic dysfunction compared to the control macrophages (Fiala et al., 2005). It has been reported that the lack of CD36, which is needed for amyloid binding and which cooperates with TLR4 and TLR6 receptors, attenuated Aβ accumulation and NLRP3 activation in macrophages (Sheedy et al., 2013). The adaptive immune system is also involved in the pathogenesis of neurodegeneration, which is demonstrated by the elevated level of Aβ-reactive T-cells in AD patients and even in healthy elderly humans and by the α-synuclein specific T-cell response in PD (Monsonego et al., 2003; Sulzer et al., 2017). In PD patients, well-characterized parts of the α-synuclein were recognized as epitopes which mostly stimulated the IL-5-expressing CD4+ cells and the cytotoxic IFNγ-producing CD8+ cells; moreover, the activation of CD4+ T-cells was accompanied by the injury of dopaminergic neurons (Brochard et al., 2009; Sulzer et al., 2017).

These results show that, after acute injuries, the immune cells are rapidly activated in order to eliminate cellular debris; however, the immune response should be mitigated in the right time, as the excessive cytokine release could promote further neuronal damage. In chronic neurodegenerative diseases, the toxic protein aggregates are generated persistently inducing a chronic immune response. Microglial and macrophage cells are assumed to clear the protein inclusions; however, during aging and in neurodegenerative diseases, their phagocytic activity declines due to cellular senescence (Luo et al., 2010; Costantini et al., 2018), while there is an increase in cytokine production. Taken together, it seems that both the lack and exaggeration of inflammatory responses can promote neuronal damage; therefore, it should be tightly regulated. In the next sections, we attempt to summarize the immunomodulatory roles of HSPs. Many evidences suggest that HSPs have several moonlighting functions through which they can be involved in the regulation of immune cell activity and cytokine release. Therefore, the modulation of HSP levels could serve as potential therapeutic applications to treat CNS diseases related to inflammation.

### Intracellular HSPs and the Heat-Shock Response

Living organisms can be subjected to environmentally constrained challenges. To survive, they have to counter these unfavorable conditions by adaptation. The term cellular stress response summarizes universal cellular reactions to a wide variety of environmental challenges (Kültz, 2005). Among the many cellular survival mechanisms, the response to heat stress is amongst the most ancient and conserved (Horváth et al., 2008). Heat stress induces multi-leveled signaling in cellular systems termed as the heat-shock response. The heat-shock response was originally defined as the biochemical response of cells to heat stress (Lindquist, 1986). Although it has been primarily described to be induced by increased temperatures, it has also been recognized that the heat-shock response can be activated by a wider range of cellular stressors like membrane perturbations, heavy metals, oxidative stress, or by pathophysiological conditions (Morimoto, 1998; Vigh et al., 2007; Balogh et al., 2013). One of the most characterized aspects of the heat-shock response is the accumulation of a conserved set of HSPs. HSPs primarily act as chaperones within the cell by aiding the folding of proteins, the refolding of denatured proteins, and by guiding the degradation of irreversibly damaged proteins. Therefore, HSPs are involved in the maintenance of protein homeostasis by alleviating the unfavorable changes caused by heat stress (Kampinga, 2006). HSPs consist of many different families differentiated by their molecular weight. In 2009, Kampinga and co-workers proposed a new nomenclature of human HSP families (Kampinga et al., 2009). Small HSPs (sHSPs) are the most diverse set of HSPs, consisting of proteins within a 12–43 kDa range. They can form multimeric complexes and display a wide range of cellular functions. The sHSPs coaggregate with aggregation-prone proteins to initiate efficient disaggregation. The release of substrate proteins from the transient sHSP reservoirs and their refolding require cooperation with ATP-dependent chaperone systems (Van Montfort et al., 2001). The sHSP holdase, HSPB1, can form large oligomeric structures which can act as molecular chaperones sequestering unfolded proteins and inhibiting subsequent aggregation and insolubilization (Singh et al., 2017). HSPB1 is additionally involved in actin polymerization/depolymerization (Tang, 2015). HSPD1 is an ATP-dependent unfoldase. The HSPD1 proteins in eukaryotes are localized to the mitochondria and are responsible for mitochondrial protein import and macromolecular assembly within this compartment (Koll et al., 1992). HSPA1 is most likely the best-characterized chaperone. The HSPA proteins act together with their many co-chaperones, working as a “HSPA chaperone machinery” (Kampinga and Craig, 2010). The most important co-chaperones are the DNAJ family and the nucleotide exchange factors. DNAJ proteins exhibit great diversity; however, all of them include a conserved J domain required for its binding to HSPA1 (Hennessy et al., 2005). DNAJ takes part in the substrate specificity of HSPA1 and can increase the ATPase activity of the HSPA1 protein thereby enhancing their activity. The nucleotide exchange factors promote the dissociation of the ADP from HSPA1. This ATPase cycle regulates the binding and release of the substrate, since HSPA proteins have a higher affinity for unfolded proteins when bound to ADP, and a lower affinity when bound to ATP (Mayer and Bukau, 2005). The HSPA1/DNAJ system participates in the active ATP-dependent folding-refolding of a high number of cellular proteins. On the other hand, the non-refoldable substrates can be selected to proteasomal degradation, which process is mediated by ubiquitin ligases as the CHIP E3 ligase (VanPelt and Page, 2017). HSPC1 is also an ATP-dependent chaperone which facilitates the maturation and/or activation of many “client proteins” involved in signal transduction and transcriptional regulation and is necessary for viability in eukaryotes. HSPC1 is central to processes broadly ranging from cell cycle regulation to protein degradation, and it has also been connected with cellular transformation (Brown et al., 2007). Therefore, the potential utilization of specific inhibitors of HSPC1 for cancer treatment has long been investigated (Schopf et al., 2017; Zuehlke et al., 2018). The proteins in the HSPH family form large hexameric structures with an unfoldase activity in the presence of ATP. These proteins are considered to function as chaperones by recessively threading client proteins through a small pore, thereby providing each client protein with a second chance to fold (Schirmer et al., 1996).

The expression of HSPs occurs under strong transcriptional control. Among eukaryotes, the transcriptional activation of HSP genes is regulated by the family of transcriptional factors termed as heat-shock factors (HSFs). The family consists of four different HSFs, from which HSF1 is the main transcriptional regulator of the HSP genes (Richter et al., 2010). HSF1 is constitutively present in its inactive form in most tissues and cell types in the cytosol. Under stress conditions, HSF1 undergoes a multistep activation process as the cytosolic HSF1 dissociates from HSPA/HSPC which prevents the interaction of HSF1 monomers. HSF1 trimerizes and subsequently translocates to the nucleus and binds to extended repeats of the sequence nGAAn, termed as heat-shock elements, in the promoter regions of its target genes (Morimoto, 1998). Moreover, in addition to activating the heat-shock response, HSF1 is involved in the regulation of many other cellular processes. HSF1 is able to modulate not only the HSP expression but the transcription of other genes as well—for example, it is involved in the regulation of apoptotic cell death, autophagy, the immune response, aging, or even synaptic function (Hooper et al., 2016; Barna et al., 2018). Several studies demonstrated that HSF1 is necessary for the normal function of the immune system, and it can regulate the expression of different inflammatory cytokines and chemokines, although its exact effect has not been fully elucidated yet (Barna et al., 2018).

HSF1 was first suggested to play a role in inflammatory processes when an abundant increase in the pro-inflammatory cytokine TNF-α was observed upon treatment with bacterial lipopolysaccharides (LPS) in HSF1 knockout mice (Xiao et al., 1999). This observation later led to the discovery of the inhibitory regulation of HSF1 over TNF-α by its binding to elements in the promoter (Singh et al., 2002), suggesting an intertwined close regulation between TNF-α signaling and HSF1. In contrast, a recent study showed that heat-shock treatment can induce the expression of TNF-α independently from NF-κB signaling. The transcription of TNF-α was activated by a multiprotein complex containing HSF1 (Ali et al., 2019), suggesting that the effect of HSF1 on TNF-α expression is likely to be influenced by other factors as well. Another level of interplay has been revealed between heat shock and TNF-mediated inflammatory signaling when TNF-α was described to cause downregulation of HSF1 activation, which was shown to increase apoptotic cell death (Schett et al., 2003). Furthermore, it has been demonstrated that heat pretreatment decreases LPS-induced IL-6 expression in macrophages and mouse embryonic fibroblast cells. The heat-induced HSF1 upregulated the ATF3 transcription factor, a negative regulator of IL-6 expression (Takii et al., 2010). This was a surprising result, as it was previously observed that HSF1 is necessary for the maximal induction of IL-6 in response to LPS treatment without heat shock (Inouye et al., 2007). Under non-stressful conditions, the HSF1 was found to bind directly to IL-6 promoter and, by promoting the opening of the chromatin structure, it may help the access of activator or repressor regulatory molecules (Inouye et al., 2007). However, the IL-6 expression of many tissues and the serum level of IL-6 were higher in HSF null-mutant mice compared to wild types after LPS injection, suggesting an inhibitory role of HSF1 on IL-6 production *in vivo* (Takii et al., 2010). Heat-shock elements can be found in the promoter region of CXC chemokine genes as well, although a heat shock has gene-specific enhancing or repressing effect on the expression of these genes (Maity et al., 2011). Generally, the increased temperature can exert diverse, both pro- and anti-inflammatory effects on macrophage function, a phenomenon that was reviewed in detail by Lee and Repasky. The authors proposed that the cytokine response induced by a mild heat treatment is largely influenced by the specific activation stage of the macrophage cells (Lee and Repasky, 2012).

It is well known that age-related processes or disease conditions can alter the cellular protein homeostasis. The accumulation of misfolded proteins increases constantly in the brain during aging, and probably, this leads to the elevation of the basal HSP level found in different brain regions (Sőti and Csermely, 2000). For example, higher level of HSPB1 and HSPA1 were detected in the olfactory bulb of middle-aged and old rats compared to young ones (Crum et al., 2015). The basal expression of HSPA1 was significantly higher in the cortex of old mice as well (Carnemolla et al., 2014). Interestingly, this study also describes a downregulation of HSPB1 in the striatum of old mice (Carnemolla et al., 2014) while others found an age-related increase in HSPB1 level in the striatum, subtantia nigra, and cortex of rats (Gupte et al., 2010; Gleixner et al., 2014). On the other hand, the heat-shock response and the inducibility of HSPs decline during aging and in disease conditions (Sőti and Csermely, 2000; Calderwood et al., 2009). Such decrease in the activation of HSF1 and thus in the stress inducibility of HSPs might appear in several conditions as it was observed during a DNA damage–initiated senescent program (Kim et al., 2012). The mouse model of HD has also been described to have a reduced heat-shock response (Neueder et al., 2017). An age-associated decrease in HSF1 activation has been observed in several studies: HSF1 activation reduced with age in rat hepatocytes (Heydari et al., 2000) and *Caenorhabditis elegans* neuronal cells also showed a significant age-associated decrease in HSF1 activation (Kern et al., 2010). Alterations in nutritional lipid supplies were also associated with a reduced heat-shock response in parallel with a marked decrease in the proportions of n-3 and n-6 polyunsaturated fatty acids (Péter et al., 2012). Also, a high-cholesterol diet–induced hyperlipidemia was described to attenuate the expression of HSPA1 following either heat or ischemic treatment in rat hearts (Csont et al., 2002). Based on these results, it is tempting to hypothesize that the impairment in HSF1 activation may not only cause a disturbed protein homeostasis but also an uncontrolled inflammatory process. Unregulated inflammation in the CNS during aging might contribute to the pathogenesis of neuronal diseases. Therefore, restoration of the heat-shock response and the HSF1 activity may help to delay age-related changes in the brain.

### HSPs and Their Role in CNS Diseases

The expression of HSPs could rapidly increase in response to different acute brain injuries, such as ischemic stroke (Sharp et al., 2013). For example, HSPA1 expression is increased in microglia, astrocytes, and endothelial cells in the infarct area, while it is elevated mainly in neurons in the penumbra (Sharp et al., 2000). In contrast, protein levels of HSPB1 are increased almost exclusively in astrocytes and to a lesser extent in certain neurons in the ischemic brain tissue following middle cerebral artery occlusion, and it is thought to stabilize the astrocyte cytoskeleton by binding to GFAP under stress conditions (Sharp et al., 2013). However, another study found that HSPB1 and HSPB5 showed a high level of expression after an ischemia/reperfusion injury and that this expression profile was mainly associated with neurons in the infarcted cortex rather than glia cells. It was also shown that HSPB1 and HSPB5 were phosphorylated after ischemia, proposing that phosphorylation can affect their neuroprotective role (Bartelt-Kirbach et al., 2017). Moreover, HSPs have been found to be associated with toxic protein accumulations in protein-misfolding neurodegenerative diseases like AD, PD, and HD. HSPB1 and HSPB5 were detected in senile plaque-associated astrocytes in the brain of patients with AD (Wilhelmus et al., 2006a). Moreover, an *in vitro* binding of HSPB1 to hyperphosphorylated tau, purified from AD brain, has also been observed (Shimura et al., 2004). HSPB5 was shown to co-localize with α-synuclein in the Lewy bodies and to strongly inhibit the elongation of the α-synuclein fibrils (Waudby et al., 2010). Swaroop et al. described an elevated expression for HSPD1 and Il-1β in a study which performed gene expression analysis on human brain samples affected with various CNS diseases such as AD, PD, stroke, or infections (Swaroop et al., 2018).

HSPs have been shown to be neuroprotective in many acute and chronic pathological conditions affecting the brain; therefore, they are considered as potential therapeutic targets (reviewed in: Tóth et al., 2015; Penke et al., 2018; Carra et al., 2019). For example, induced expression of HSPA1 in APP/PSEN1 mice decreased the level of inflammatory cytokines (Sun et al., 2017), whereas transgenic overexpression of HSPA1 or HSPB1 in mouse models of AD was able to reduce Aβ plaque formation and cognitive dysfunctions (Hoshino et al., 2011; Tóth et al., 2013) In addition, HSPB8 can promote motor neuron survival by diminishing the toxic effects of dipeptide repeat proteins (Cristofani et al., 2018; Zhu and Reiser, 2018). Current studies revealed that the inhibition of HSPC1 can ameliorate the symptoms of neurodegenerative diseases through inducing the activation of HSF1 and HSPA1, as well as the clearance of protein aggregates (Thirstrup et al., 2016; Wang et al., 2017). Elevated levels of small HSPs are able to ameliorate neuronal cell death under acute brain-damaging conditions as well, like cerebral ischemia and ethanol administration (Tóth et al., 2010; van der Weerd et al., 2010; Arac et al., 2011). Furthermore, HSPA1 overexpression improved the outcome of stroke by preventing neuronal apoptosis (Kim et al., 2016).

As molecular chaperones, the main role of HSPs is to suppress the aggregation of misfolded toxic proteins, which can explain their protective effects in protein-misfolding disorders, like AD or PD. HSPB1 and HSPB5 were found to bind to Aβ, inhibiting its fibril formation, therefore reducing its cytotoxic effect in cerebrovascular cell culture *in vitro* (Wilhelmus et al., 2006b). HSPB1 and HSPB5 were also shown to interact with the aggregation-prone α-synuclein to prevent its aggregation *in vitro* (Cox et al., 2016). Baughman et al. revealed that both HSPB1 and HSPA8 are able to prevent the formation of toxic tau fibrils. HSPB1 can transiently interact with tau during the early phase of fibril formation, while HSPA8 binds to tau tightly at later stages (Baughman et al., 2018). This data indicates that these HSPs might complement each other’s function under stress conditions. To restore protein homeostasis, HSPs can also aid the degradation of misfolded proteins *via* the ubiquitin-proteasome system or by autophagy, to prevent their accumulation in the brain. According to earlier results, under ischemia-induced hypoxia, the level of chaperone-mediated autophagy, which specifically degrades the HSPA8 target proteins, was enhanced which was beneficial for cell survival (Dohi et al., 2012). Another study describes HSPB8 and HSPA8 as part of a multi-protein complex responsible for selectively removing aggregated proteins in amyotrophic lateral sclerosis (Crippa et al., 2010). It was demonstrated that the collaboration between HSPB8 and the co-chaperone BAG3 can enhance autophagy and thereby increase the clearance of protein inclusions produced in motor neuron diseases (Crippa et al., 2016).

Aside from their classical chaperone function, it has been shown that HSPs have multiple functions within a cell (Horváth et al., 2008; Lanneau et al., 2008; Miller and Fort, 2018). In addition to their central role in the regulation of protein homeostasis, HSPs may exert neuroprotection through several non-chaperone functions (Tóth et al., 2015). For example, neuronal cultures from the hippocampus of mice overexpressing HSPB5 were shown to be able to sustain their dendritic structures upon heat shock (Bartelt-Kirbach et al., 2016) suggesting a role in preserving the dendritic structure and neuronal connectivity upon stress. Numerous data confirm that HSPs can also protect cells against oxidative stress, which is increased in acute and chronic neurodegenerative conditions. This function of HSPs seems to be independent of their anti-aggregation effect in certain cases (Wyttenbach et al., 2002). Moreover, HSPs have been demonstrated to inhibit apoptotic cell death cascades triggered by toxic, aggregation-prone proteins, like α-synuclein or Aβ (Zourlidou et al., 2004, Yurinskaya et al., 2015). HSPs can also exert their beneficial role through the protection of the BBB, the damage of which was associated with many CNS diseases (Sweeney et al., 2019). HSPB1 expressed in endothelial cells prevented the BBB disruption and immune cell infiltration by stabilizing the junctional proteins and inhibiting actin polymerization after ischemia (Shi et al., 2017). Moreover, phosphorylated recombinant HSPB1, which was administered intravenously after ischemic injury, was also found to be effective in preventing severe BBB damage (Shimada et al., 2018).

All these activities of HSPs could have an alleviating effect on the pro-inflammatory milieu associated with neurological diseases. By the reduction of neuronal cell death and protein aggregation, HSPs might preclude the initiation of inflammation. By preserving the integrity of the BBB, they might constrain the infiltration of peripheral immune cells. However, an emerging number of evidence support that HSPs, especially extracellular HSPs, directly interact with immune cells. The next chapter of the review is aiming to describe this phenomenon in more detail.

### Extracellular HSPs

Historically HSPs have been regarded as intracellular molecules but, in the last decades, it was revealed that they can also occur in the extracellular space (reviewed in: van Noort et al., 2012; Giuliano et al., 2011; De Maio and Vazquez, 2013; Calderwood, 2018; Reddy et al., 2018). Many different cell types have been shown to be able to secrete HSPs. The first studies describing extracellular HSPs were performed on squid glial cells (Tytell et al., 1986) and rat embryonal cell cultures (Hightower and Guidon, 1989). Tumor cells also secrete high amount of HSPA5 (Kern et al., 2009), HSPC3 (Suzuki and Kulkarni, 2010), or HSPB1 (Stope et al., 2017), and HSPB5 was detected in the media of cultured human adipocytes as well (Lehr et al., 2012). Thermal stress has been demonstrated to increase the amount of secreted HSPA1 from astrocytes (Taylor et al., 2007) and HEPG2 cells (Vega et al., 2008). Moreover, a recent study showed the release of phosphorylated HSPB1 from aggregating human platelets (Tokuda et al., 2018). Therefore, we can suppose that stress protein release may have important regulatory roles under physiological and pathological conditions (Calderwood et al., 2007). One possible mechanism of HSP release is the lysis of damaged, necrotic cells. Several HSPs such as HSPA1, HSPC1, DNAJ, or HSPB1 were found in the supernatant of E.G7 mouse T-lymphocytes (Basu et al., 2000) or HeLa cells (Saito et al., 2005) after the induction of necrotic cell death by freeze-thaw cycles. First, it was assumed that this is the only way for HSPs to be released from a cell. If we suppose that necrotic cells are the main source of extracellular HSPs, the presence of HSPs in the extracellular environment would indicate tissue damage. However, this cannot explain the selective release of HSPs even under non-lethal, mild stressful conditions when cell death is not significantly present. For example, a physiological, fever range heat treatment was able to induce HSPA1 release from prostate carcinoma cells (Mambula and Calderwood, 2006). Cardiac myocytes secrete HSPD1 even under unstressed conditions; however, a mild hypoxic stress further increased the amount of HSPD1 released without signs of necrotic cell death (Gupta and Knowlton, 2007). Similarly, HSPA1 was secreted by purified human peripheral blood mononuclear cells under normal culture conditions and after heat treatment with a barely detectable cell damage (Hunter-Lavin et al., 2004). The mechanism of active, controlled release of HSPs is unclear as they have no secretion leader signal in their sequence and inhibitors of the classical protein transport, and secretory pathways cannot prevent HSP secretion. For example, brefeldin A which is able to block protein transportation from the endoplasmic reticulum to the Golgi seemed to be ineffective in arresting HSP release either in the above mentioned studies (Hunter-Lavin et al., 2004; Gupta and Knowlton, 2007) or in heat-shock-treated Caco-2 cells (Broquet et al., 2003). In contrast, HSPA1 release was inhibited by methyl-β-cyclodextrin, a chemical that has membrane raft-disrupting effect suggesting that the association of HSPA1 with lipid rafts is necessary for its secretion (Broquet et al., 2003; Hunter-Lavin et al., 2004). Indeed, several non-traditional secretory mechanisms were proposed to explain the regulated release of HSPs. Methylamine treatment has been demonstrated to block HSPA1 export in different cell types. As this compound can increase intra-lysosomal pH, it was proposed that the HSP secretion might be mediated by secretory lysosomes (Hunter-Lavin et al., 2004; Mambula and Calderwood, 2006). This was also supported by the observation that heat shock stimulates lysosomal exocytosis and increases the HSPA1 level in the lysosomal fraction (Mambula and Calderwood, 2006). Co-localization of HSPB1 with a lysosomal marker suggests that this sHSP can also be secreted through the lysosomal pathway (Rayner et al., 2008; Lee et al., 2012a). As the inhibition of exosomal release from cultured cells was shown to inhibit the secretion of HSPs, exosomes can be considered as another potential carriers for extracellular HSPs (Gupta and Knowlton, 2007; Rayner et al., 2009). These small cell-derived vesicles are generated constitutively by many cell types, like immune cells; however, their release is further increased in response to cellular interactions or stress conditions. By transferring lipids, cytosolic proteins, or RNAs from the donor cells, these vesicles can influence the function of acceptor cells; therefore, they have important roles in cell–cell communication (Campanella et al., 2014; Edgar, 2016). Until today, many studies confirmed the presence of different HSPs in exosomal vesicles released by cancer cells (Chalmin et al., 2010; McCready et al., 2010; Stope et al., 2017), primary cortical astrocytes (Nafar et al., 2016), or by human retinal pigment epithelial cells (Sreekumar et al., 2010). However, it still remains unclear if HSPs are only cargo materials in exosomes or they also can act as targeting molecules toward the host cells by being localized on the exosomal surface. When HSPs were observed to be present only in the lumen of exosomes, there was no observable interaction between them and the surface receptors of the target cell (Clayton et al., 2005). In this case, the exosome-delivered HSPs cannot interact directly with the target cells only if the vesicles “burst” outside of the cell or fuse with the cell membrane, releasing HSPs into the extracellular space or into the cytosol, respectively (De Maio and Vazquez, 2013). On the other hand, other studies found HSPs in a membrane-bound state in exosomes. For example, HSPA1 has been demonstrated to leave heat-shock-treated HEPG2 cells in a membrane-associated form (Vega et al., 2008). Also, in primary rat cortical astrocytes, Aβ induced the exosomal release of HSPB1, which was associated with the membrane of the exosomes (Nafar et al., 2016). Tumor cell–derived exosomes were found to express HSPA1 on their surface (Gastpar et al., 2005; Chalmin et al., 2010) which makes it possible to stimulate the target cells through surface HSP receptors.

Therefore, it seems that HSPs are released continuously even from unstressed cells. Under stress conditions, cells respond to injury with a strong increase of HSP expression to cope with stress-induced impairments like protein denaturation or apoptosis. At the same time, cellular stress promotes a subsequent release of HSPs into the extracellular space (Calderwood et al., 2007). Accordingly, many reports demonstrated the increased level of HSPs in body fluids after different stress insults and pathological conditions (Quintana and Cohen, 2005; De Maio and Vazquez, 2013; Reddy et al., 2018). HSPD1 levels in the serum are increased in both temporal lobe epilepsy patients and in the animal model of the disease in response to seizures, suggesting that plasma HSPD1 can be used as a biomarker reflecting neuronal cell death (Gammazza et al., 2015). Elevated serum HSPD1 is also related to the severity of both acute and chronic heart failures (Bonanad et al., 2013). HSPA1 level in the blood seems to be a useful biomarker for the detection of different tumor types such as pancreatic cancer (Dutta et al., 2012) as well as head and neck squamous cell carcinoma (Gehrmann et al., 2014). Moreover, the serum level of HSPA1 is increased in diabetic patients and correlates with the severity of the disease (Nakhjavani et al., 2010). Extracellular small molecular weight HSPs have also been associated with different pathological states, as HSPB1 has been detected in the serum of patients with several types of cancer, such as breast cancer (Fanelli et al., 1998; Banerjee et al., 2011), hepatocellular carcinoma (Feng et al., 2005), gastric adenocarcinoma (Huang et al., 2010), colon cancer (Thuringer et al., 2015), or pancreatic carcinoma (Melle et al., 2007; Liao et al., 2009). Plasma HSPB1 level is increased in chronic pancreatitis (Liao et al., 2009), and during acute ischemic stroke conditions (Gruden et al., 2013). Myocardial cells were further observed to release HSPB1 after ischemia (Jin et al., 2014). Lesion size after stroke was found to be strongly correlated with serum HSPB5 levels in a human study, however, interestingly only in younger patients, suggesting that this response is age-dependent (Arac et al., 2011). Extracellular HSPs which are released from infected, damaged, or stressed cells are able to trigger immune response by activating many cells of the innate and adaptive immune systems and can act as local “danger signals” that promote stress response programs in surrounding cells.

### Heat-Shock Proteins and Inflammation Modulation

Extracellular HSPs were shown to have a relatively distinct role from what was observable as their intracellular action. Although, the transmission of HSPs between cells was shown to contribute to the maintenance of normal proteostasis under non-stress conditions (Takeuchi et al., 2015), HSPs are released more substantially from cells under stress conditions. The first observation describing the presence of extracellular HSPs was already associated with stress when a distinct transfer of HSP like proteins was described from glial cells to the axon of neuronal cells upon heat stress, in a squid model system (Tytell et al., 1986). This study was also among the first to suggest a neuroprotective role of stress proteins in the nervous system. Later, this phenomenon was shown to be true for mammalian cells because human glioblastoma cells were also shown to secrete a large amount of HSPA1, and human neuroblastoma cells were described to take up exogenously added, purified HSPA1 which conferred the neuronal cells with an elevated resistance against heat stress and apoptosis (Guzhova et al., 2001). Evidence from studies into intracellular HSPA1 points toward the conclusion that intracellular HSPA1 can exert an anti-inflammatory effect in the brain. A study conducted with mice overexpressing HSPA1 showed that, during ischemic conditions, HSPA1 could exert an anti-inflammatory action by the reduction of NF-κB activity (van Eden et al., 2005). Another study also suggested a similar action for HSPA1 during ischemia as it was able to interfere with the activation of NF-κB by inhibiting the phosphorylation IκB by IκB kinase (IKK) (Zheng et al., 2008). This was also associated with the reduction of microglia-mediated cell death of astrocytes. Moreover, decreased DNA-binding activity of NF-κB was found in an HSPA1 overexpressing mouse microglial cell line in response to TNFα treatment (Sheppard et al., 2014). In contrast to the notion that HSPB1 is traditionally viewed as a chaperone responsible for acting as a holdase to hand over destabilized proteins for the ATP-dependent chaperone system, intracellular HSPB1 also provides protection against apoptosis by inhibiting mitochondrial apoptotic pathways and by facilitating the activation of NF-kB (Concannon et al., 2003; Guo et al., 2009). In microglial cells, HSPB1 was shown to inhibit the activation of NF-kB and decrease microglial TNF-α production by promoting the lysosomal degradation of IKKβ by chaperone-mediated autophagy (Liu et al., 2018b). HSPB1 was also associated with an intracellular anti-inflammation modulatory function in epithelial cells, where HSPB1 was able to associate with the IKKβ and IKKα and inhibit their activity thereby suppressing NF-κB activation (Kammanadiminti and Chadee, 2006). This evidence implies that extracellular HSPs, after being taken up by the target cell, may act through a similar mechanism which would confer resistance to recipient cells in the brain. As mentioned previously, extracellular vesicles may fuse with the membrane of target cells, releasing the HSPs which are in their lumen into the cytosol of the target cell (De Maio and Vazquez, 2013). Therefore, it can be assumed that, similarly to intracellular ones, HSPs from extracellular origin might have neuroprotective, anti-apoptotic, and anti-inflammatory effects.

On the other hand, extracellular HSPs, either released passively from necrotic cells or secreted actively and presented on the surface of extracellular vesicles, can bind to several receptors of the target cells, influencing immune functions. Indeed, many *in vitro* studies confirmed that the treatment of different cell types with exogenous HSP preparations has an impact on the cytokine response. HSPA1 treatment of LPS-activated monocytes decreased the level of TNF-α and IL-6 (Ferat-Osorio et al., 2014). Interestingly, addition of extracellular HSPA1 to bone marrow–derived murine dendritic cells resulted in a reduced basal level of TNF-α and IFN-γ (Borges et al., 2013). In contrast, other evidence implies an opposite pro-inflammatory role for extracellular HSPA1 as it has been described to promote inflammation by the activation of NF-κB and the stimulation of human macrophages to secrete pro-inflammatory cytokines TNF-α, IL-1β, and IL-6 (Asea et al., 2000). Exogenous HSPA1 can increase the production of IL-6 and TNF-α in a concentration-dependent manner in rat microglial culture as well (Kakimura et al., 2002). Moreover, extracellular HSPA1 and HSPC1 are involved in the activation of the adaptive immune response. By sequestering antigenic molecules, they can help their transfer to antigen presenting cells and their load onto MHC molecules (reviewed by Calderwood et al., 2007; van Noort, 2008; Zininga et al., 2018). Compared to HSPA1, the functions of other extracellular HSPs, like sHSPs, are relatively less well defined despite the fact that they are detected to be present in the extracellular environment. Among these proteins, sHSPs are intriguing targets to study in the neurobiological context since they have already been implicated to act as neuroprotective agents in several conditions intracellularly. Considering that HSPB1 has been associated with the transcription factor NF-kB in many studies, it is tempting to speculate that, among the sHSPs, HSPB1 could exert an extracellular control over inflammatory processes in the brain. The extracellular release of HSPB1 has been suggested to have a role in modulating the immune response around the cell, as extracellular HSPB1 was found to activate NF-κB in macrophages (Salari et al., 2013) and myocardial cells (Jin et al., 2014). HSPB1 has been demonstrated to increase the level of IL-8 while inhibiting the production of TGF-β1 CD40 ligand in astrocytes *in vitro* (Bruinsma et al., 2011). The treatment of microglia cultures with α-crystallin resulted in the activation of the cells and increased the production of nitric oxide and TNFα (Bhat and Sharma, 1999). On the other hand, another study showed that α-crystallin pretreatment can reduce the level of the same molecules induced by LPS in primary rat microglial cell culture (Wu et al., 2009). Moreover, it effectively reduced astrocyte activation *in vitro*, in response to LPS treatment (Masilamoni et al., 2006).

**Figure 2:**
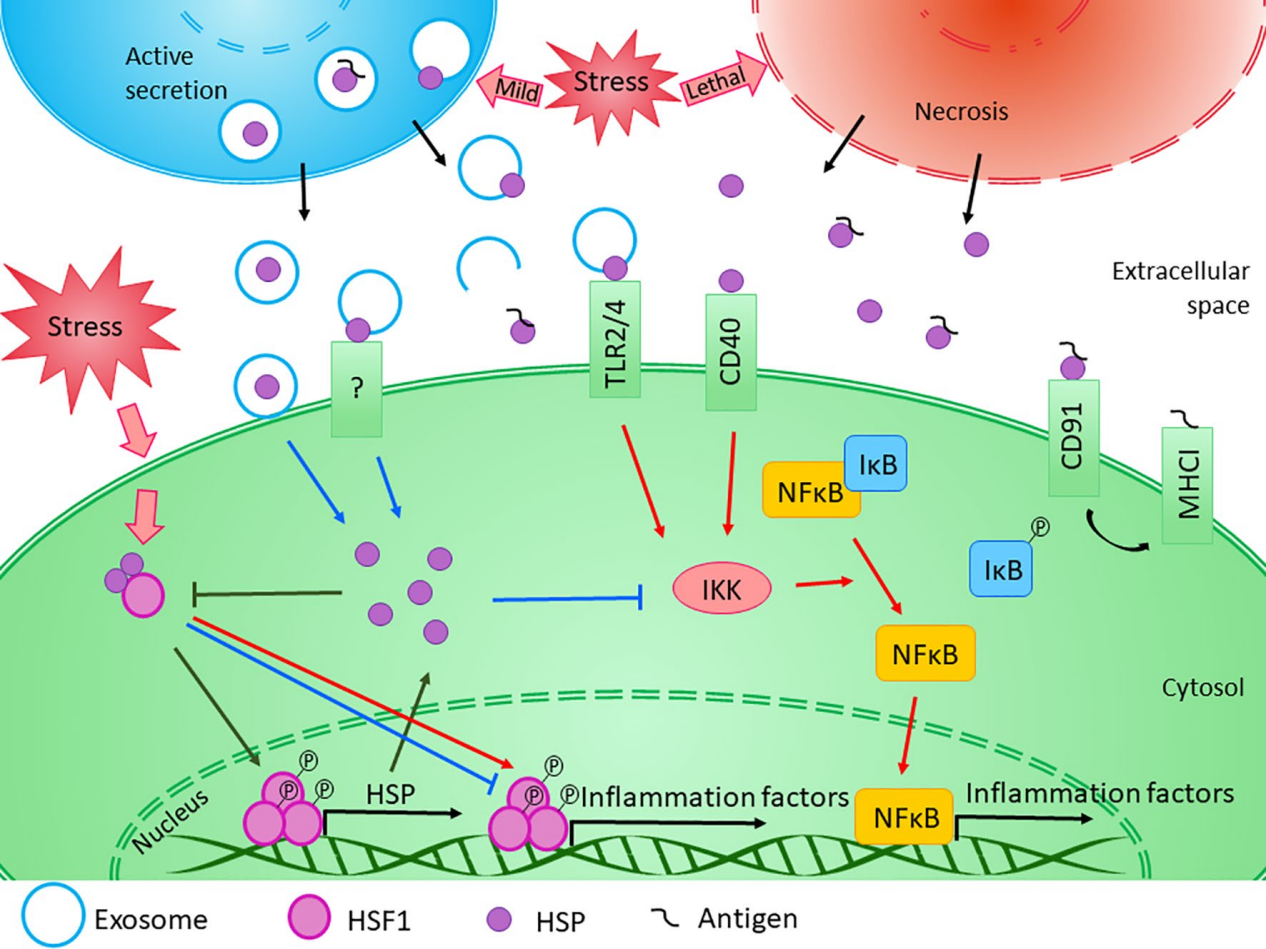
Diverse effects of HSPs on inflammation. The exact role of HSPs in the regulation of innate and adaptive immune responses is influenced by several factors. Transcription of HSP genes is regulated by HSF1, which normally presents in the cytosol in its inactive form bounded to HSPs. Under stress conditions, the monomeric HSF1 dissociates from HSPA/C, trimerizes, and subsequently translocates to the nucleus, where it upregulates the expression of HSPs. Moreover, HSF1 was suggested to have bi-directional effect on the expression of inflammatory factors. HSF1 was shown to inhibit the expression of TNF-α by binding to its promoter, while it was also able to activate TNF-α as part of a multiprotein complex. Similarly, HSF1 was found to bind directly to IL-6 promoter opening of the chromatin structure thereby facilitating the access of activator or repressor molecules. The newly synthesized intracellular HSPs have protective and anti-apoptotic effects. Both HSPB1 and HSPA1 were shown to inhibit the activation of NF-κB by inhibiting IKK, thereby exerting an anti-inflammatory function. At the same time, cellular stress promotes the release of HSPs into the extracellular space. Lethal stress leads to the passive secretion of HSPs from necrotic cells, which usually induce a strong pro-inflammatory activity. On the other hand, HSPs can be secreted actively even under non-lethal, mild stress conditions. Although the classical protein secretory pathways are not secreting HSPs, alternative secretory mechanisms, such as exosomal release can be the basis of their regulated export. HSPs were found in the lumen of exosomal vesicles or they can occur in a membrane-associated form. Luminal HSPs cannot interact directly with the target cells only if the vesicles “burst” outside of the cell or fuse with the cell membrane, releasing HSPs into the extracellular space or into the cytosol. HSPs presented in the membrane of extracellular vesicles can bind to various surface receptors, such as toll-like receptors, that can induce the NF-κB pathway. Moreover, by sequestering antigenic molecules, HSPs are involved in antigen presentation. On the other hand, many studies confirmed that exogenous HSPA1 and HSPB1 exert anti-inflammatory effects through yet undefined mechanism in different pathological conditions. (Reviewed in van Noort, 2008; De Maio and Vazquez, 2013; Kim and Yenari, 2013). Red arrows denote pro-inflammatory, while blue arrows denote anti- inflammatory processes.

These results clearly show that HSPs are involved in the regulation of immune responses in different organs, including the brain. However, whether their exact effect is anti- or pro-inflammatory, it depends on many factors (**Figure 2**). An interesting observation came from by Basu et al. that necrotic but not apoptotic cells are able to release HSPA1, HSPC1, and calreticulin which were found to be immune-stimulatory through the activation of antigen-presenting cells *via* the NF-κB pathway (Basu et al., 2000). This result suggests that the effect of the released HSP is highly dependent on the state of the donor cell and the cellular processes taking place therein. For example, as described in the “extracellular HSP” section, aside from the active vesicular secretion, HSP could be excreted passively, by necrosis. The emergence of the seemingly contradicting observations describing HSPA1 both as a pro- and an anti-inflammatory agents have led to the hypothesis that the context as to how HSPA1 (and possibly other HSPs) reaches the extracellular environment matters in regard to their modulatory action on inflammation (Broere et al., 2011; van Eden et al., 2012). However, probably not the donor cell’s state is the only factor that can influence the effect of HSPs. Similarly, as was proposed in the case of heat treatment (Lee and Repasky, 2012), we can suppose that the HSPs themselves might have diverse effects based on the status of the recipient cell.

Moreover, several receptors have been characterized for HSPs which can all turn on specific signaling pathways. This suggests that the anti- or pro-inflammatory roles of HSPs are strongly dependent on their cell surface receptors as well. HSPs exert their immunomodulatory functions on macrophages, dendritic cells, T cells, B cells, and NK cells *via* many different receptors (reviewed in Quintana and Cohen, 2005; Calderwood et al., 2016). For example, CD91, CD40, and the chemokine receptor CCR5 have been demonstrated to interact with HSPA1 (Basu et al., 2001; Becker et al., 2002; Pido-Lopez et al., 2007), of which CD40 and CCR5 are expressed on the surface of microglial cells. Scavenger receptors such as LOX-1 and SREC-1 can act as receptors not only for HSPA1 but for HSPD1 and HSPC1 as well (Thériault et al., 2006; Murshid et al., 2010; Xie et al., 2010a). Two members of the TLR family, namely, TLR 2 and TLR4, are considered as HSP receptors; however, their specific interactions have hitherto been questioned. As the same receptors are responsible for the detection of bacteria-derived danger signals, like LPS, it seems to be possible that the cytokine induction was not caused by the HSPs themselves rather by the co-purified bacterial endotoxins (reviewed in Quintana and Cohen, 2005; van Noort, 2008; Kim and Yenari, 2013). However, later several studies demonstrated that non-bacteria-derived HSP preparations can also induce macrophage activation (Basu et al., 2000; Wang et al., 2006; Vega et al., 2008). These studies also draw attention to the probability that sequestering of different molecules by HSPs could play a role in their physiological immunomodulating effect. HSPs are able to bind to a broad array of molecules from bacterial LPS to tumor-derived peptides, augmenting their immunogenicity (Quintana and Cohen, 2005). This was confirmed by a study demonstrating that HSPD1 and HSPA1 alone were inefficient to induce the TNF-α secretion of human peripheral blood–derived mononuclear cells; however, they can potentiate LPS-induced TNF-α release when added 24h prior to the stimulation (Bangen et al., 2007). It is likely, that other, yet uncharacterized receptors exist through which the immunomodulatory effects of HSPs could be mediated. Furthermore, the combinatorial effect of the different cell surface receptors might influence the exact pro-or anti-inflammatory role of certain HSPs (Calderwood et al., 2016).

It is also important to note that based on changes in the amount of a single cytokine, it is not possible to determine clearly if a process is pro- or anti-inflammatory. For example, TNF-α seems to have a dual role in neuroinflammation and can be involved in both pro- and anti-inflammatory response profiles (van Noort et al., 2012). Therefore, measuring the levels of only a few cytokines in a certain isolated cell line is probably not sufficient to determine the overall biological effect of a treatment (van Noort et al., 2012). The situation is further complicated by the fact that the inflammatory process itself may have beneficial and harmful outcomes as well. Therefore, to determine the role of HSPs in inflammatory brain diseases, we should take into consideration several symptoms not only the level of inflammatory factors.

Results suggest that exogenously added HSPs have a general anti-inflammatory effect in different acute brain injuries. Several evidences support the potential neuroprotective role of extracellular HSPA1 since it was described to inhibit motor and sensory neuronal cell deaths after traumatic nerve injury (Tidwell et al., 2004) and to preserve synaptic transmission during hyperthermic conditions (Kelty et al., 2002). The exogenous addition of HSPA8 was also shown to prevent axotomy-induced death of sensory neurons (Houenou et al., 1996). The injection of recombinant HSPB1 exerted a protective effect against ischemic injury and decreased inflammation in the brain during these processes (Shimada et al., 2018). Apart from HSPB1, another sHSP, HSPB5 was also described to have an anti-inflammatory role when applied extracellularly whereby it acted against pathologic CNS inflammation in autoimmune demyelination and ameliorated experimental autoimmune encephalomyelitis in mice (Ousman et al., 2007). Intraperitoneal injection of recombinant HSPB5 was able to reduce lesion size and modulate the peripheral inflammatory response, even when the treatment was started 12 h after stroke onset (Arac et al., 2011). HSPB4/B5 isolated from bovine lens prevented the elevation of GFAP and NF-κB expression induced by acute inflammation in the brain of silver nitrate–treated mice (Masilamoni et al., 2006). Intravitreal injection of α-crystallin decreased the expression of TNFα and iNOS after optic nerve injury *in vivo* in rats (Wu et al., 2009). In contrast, the intrathecal injection of HSPD1 activated the TLR4 pathway which resulted in neurodegeneration and demyelination (Rosenberger et al., 2015). Furthermore, another microglial receptor, LOX-1, was described to bind to HSPD1 and to promote a deleterious inflammatory process (Zhang et al., 2012). HSPD1 is originally localized to the mitochondria but, in the case of brain injury, it was also found to be present in the extracellular space. This extracellular presence is due to the fact that, during CNS disorders, mitochondria dysfunction occurs, leading to the abnormal distribution of HSPD1 (Cappello et al., 2010). The extracellular HSPD1 can serve as a danger signal, and in cell cultures, it was shown to induce neuronal damage which was associated with the TLR4-MyD88 signaling pathway in microglia cells (Lehnardt et al., 2008).

Numerous evidences support the protective role of HSPs in chronic neurodegenerative diseases *in vivo*, especially in protein-misfolding disorders, described briefly in the section “HSPs and their role in CNS diseases.” However, most of these studies were performed on transgenic animals overexpressing different HSPs, or the increased expression of HSPs was induced by drug treatment. Therefore, it is difficult to separate the specific effects of intra- and extracellular HSPs, although one can suppose that both are involved in ameliorating the symptoms. For example, in a Drosophila model, it was demonstrated that HSPA, secreted into the extracellular space, can bind to Aβ reducing its neurotoxic effect and therefore the level of cell death (Fernandez-Funez et al., 2016). In contrast, it is difficult to find studies in which exogenously added HSPs are used to treat chronic neurodegenerative diseases in animal models. Nonetheless, several results suggest that the neuroprotective effect of HSPs is partly based on their ability to influence the function of immune cells. Microglial cells activated by exogenous HSPA1 or HSPC1 showed an elevated capacity for Aβ phagocytosis suggesting that extracellular HSPs might facilitate the clearance of these misfolded proteins (Kakimura et al., 2002). This effect of HSPC1 was confirmed later *in vivo* as well, by the simultaneous intra-hippocampal injections of Aβ and HSPC1 (Takata et al., 2003). HSPD1 was also found to stimulate phagocytosis in the N9 murine microglial cell line. Interestingly, this effect was diminished by the downregulation of TREM2 receptor. As HSPD1 was found to bind to TREM2, it is likely that the HSPD1-induced activation of phagocytosis might be mediated through this receptor (Stefano et al., 2009). As the decline of the normal phagocytic activity might have an important role in the pathogenesis of diseases associated with protein misfolding (see details in “Role of neuroinflammation in different acute and chronic CNS diseases” section), the promotion of the engulfment of toxic proteins by microglial cells might be useful in the treatment of these diseases. Therefore, HSPs could be important therapeutic targets in both acute and chronic neurological diseases, not only because of their classical chaperone function, but also considering their ability to modulate inflammatory processes.

### Modulation and Application of Heat-Shock Proteins as Potential Therapies Against Chronic Neuroinflammation

Based on the accumulated evidence reviewed herein, HSPs themselves and compounds modulating the heat-shock response could potentially open up new avenues for inflammation modulation in the nervous system. Approaches to fine-tune the intracellular amount of HSPs and to deliver functional, therapeutic extracellular HSPs to cells are on the rise and would certainly contribute to the panel of medical treatments against inflammation-associated diseases in the nervous system. For this purpose, membrane lipid therapy seems to be a promising solution, as membrane fluidity and microdomain organization are involved in the regulation of the cellular heat-shock response. For example, manipulating membrane lipid composition can influence the release, and therefore the immune stimulatory effects, of HSPs (Vigh et al., 2007). An emerging number of studies are describing the manipulation of HSPs by chemical means (Török et al., 2014). HSP co-inducers were already successfully applied in mice against hypoxia-induced brain damage (Xu et al., 2011), chemotherapy-induced peripheral neuropathy in rats (Bárdos et al., 2003), and were shown to have neuroprotective effects in a mouse model of AD (Kasza et al., 2016). Arimoclomol, a drug which acts like an HSP co-inducer, was suggested as a candidate for the therapy of amyotrophic lateral sclerosis as it can improve hind limb muscle function and motoneuron survival in a mouse model of the disease (Kieran et al., 2004; Phukan, 2010). Arimoclomol was also been demonstrated to have a beneficial effect against the neuronopathic form of Gaucher disease, by the induction of the HSPA5 (Fog et al., 2018). The pharmacological enchantment of the unfolded protein response and the heat-shock response by arimoclomol also provided protection against retinal degeneration in the animal model of rhodopsin retinitis pigmentosa (Parfitt et al., 2014). It is important to note that arimoclomol was described to be non-toxic in a double-blind, placebo-controlled trial in which patients with amyotrophic lateral sclerosis received the drug (Benatar et al., 2018). BRX-220, another HSP co-inducer, was shown to protect motoneurones from axotomy-induced cell death and affect the upregulation of HSPC1 and HSPA1 in parallel, in glia and neuron cells (Kalmar et al., 2002). The same compound was also observed to be effective in the prevention against peripheral neuropathy (Kürthy et al., 2002) and to promote restoration of morphological and functional properties in the sensory system following peripheral nerve injury (Kalmar et al., 2003).

Geranylgeranylacetone, an HSP inducer, was demonstrated to alleviate cerebral ischemic damage in the brains of rats (Uchida et al., 2006). Similar observations were made with respect to α-lipoic acid, a heat-shock response activator, in the gerbil brain following ischemic stress (Lee et al., 2012b). Pharmacological induction of HSPA1 in microglia and neurons by 17-N-allylamino-17-demethoxygeldanamycin (17-AAG) was shown to reduce the volume of hemorrhage and improve neurobehavioral outcomes in a mouse model of experimental traumatic brain injury (Kim et al., 2015). Another HSP inducer celastrol, a chemical compound isolated from the *Celastracea* family of plants, was identified to induce the phosphorylation and DNA binding of HSF1, which consequently can result in the upregulation of several HSPs (Westerheide et al., 2004). The fact that celastrol can activate heat-shock gene transcription alone or synergistically with other stresses or compounds makes it a promising adjuvant drug candidate (Westerheide et al., 2004). Celastrol treatment was shown to increase the expression of HSPA1 and reduce the levels of TNF-α in spinal motor neurons and contribute to motor neuron survival in the SOD1(G93A) transgenic mouse model of amyotrophic lateral sclerosis (Kiaei et al., 2005). Other drugs with herbal origins have been also shown to act like HSP inducers. Paeoniflorin, a monoterpene glycoside, was characterized to induce HSP through the activation of HSF1 and the formation of HSF1 containing granules in the nucleus (Yan et al., 2004). The treatment of rats with paeoniflorin can protect the brain from cerebral ischemic injury *via* inhibition of apoptosis (Zhang et al., 2015). Glycyrrhizin a triterpenoid saponin was also suggested to be an HSP co-inducer (Yan et al., 2004). Several other compounds have been known to have a positive effect on HSP expression such as the hydroxylamine derivative HSP co-inducer bimoclomol (Vígh et al., 1997), the antiulcer drug geranylgeranylacetone (Hirakawa et al., 1996) or the benzoquinone antibiotic herbimycin A (Morris et al., 1996). All of which were being effective against different pathophysiological conditions in the CNS, as bimoclomol proved to be cytoprotective for retinal glia and neuronal cell against diabetes-related ischemic cell damages (Bíró et al., 1998), while geranylgeranylacetone is described to be neuroprotective against traumatic brain injury (Zhao et al., 2013), and herbimycin A treatment was capable to induce sympathetic neuron survival and to protect against hypoxia-induced neuronal damage (Cabral-da-Silva et al., 2003).

Aside from the pharmacological modulation of their intracellular expression levels, HSPs themselves can be used as therapeutic agents, as described by several studies. Purified HSPA1/HSPA8 proteins injected into the third ventricle of the brain were found to infiltrate into the cytosol of the neurons in different brain regions. In parallel, the treatment was able to decrease the duration and severity of chemically induced seizures and reduce muscle tonus (Ekimova et al., 2010). Similarly, intranasally injected human HSPA1 rapidly penetrate into the brain showing mainly intracellular localization (Bobkova et al., 2014). Furthermore, exogenous recombinant HSPA1 ameliorates symptoms of AD such as Aβ accumulation and memory deficits (Bobkova et al., 2014) and improves cognitive functions and increases lifespan of old mice (Bobkova et al., 2015). The administration of recombinant HSPA1 to glioblastoma cells within the brain of rats has also shown to alter the inflammatory environment within the surrounding region of the tumor as it has induced the activation and IFNγ-secretion of CD8+ cells (Shevtsov et al., 2014). These studies are highlighting the therapeutic potential of the exogenous delivery of recombinant HSPs into the nervous system. However, the delivery and targeting of these molecules into the specific brain regions could present a significant challenge.

The aforementioned and well-characterized interaction of HSPs with membranes (Horváth et al., 2008) might open up new possibilities to come across the biological barriers preventing the efficient delivery of biological molecules to the brain with a goal to achieve the beneficial modulation of inflammatory processes. Several HSPs were found to interact and be inserted into the plasma membrane, as well the release of HSPs to the extracellular environment is associated with membranes (Balogi et al., 2019). Thus, it is plausible that the therapeutic application of membrane-bound HSPs could significantly enhance their anti- or pro-inflammatory activity. HSPA1 was described to be released into the extracellular environment upon heat stress in a membrane-associated form originating from the plasma membrane also the membrane-bound HSPA1 showed a significantly higher potential to induce TNF production in macrophages compared to the free recombinant form of the protein (Vega et al., 2008). Similarly, other observations have detected that exosome containing HSPA1 on their surface can stimulate the migration and cytolytic activity of NK cells (Gastpar et al., 2005). Membrane-bound HSPA1 was also associated with the stimulation of a type 1 CD4(+) helper T cell and a CD8(+) cytotoxic T lymphocyte response (Xie et al., 2010b).

Extracellular membranous vesicles as exosomes have already been associated with neuroinflammation (Gupta and Pulliam, 2014). The liposomal delivery of protein into the nervous system has also recently been utilized to alleviate inflammatory processes (Vieira and Gamarra, 2016). However, the insertion of proteins to the bilayer is presenting a significant challenge during the preparation of liposomes. This problem can be circumvented by the application of a novel form of nanoparticles termed as membrane nanodisks, where a lipid bilayer is surrounded by an amphipathic protein belt (Rouck et al., 2017). Membrane nanodisks are also showed to be potential vehicle for CNS delivery, as it has been described to cross the BBB and to effectively target glioma cells within the brain (Wang et al., 2018b). The application of these membrane nano-carriers might provide a useful strategy to deliver membrane-bound HSPs to the nervous system. The use of membrane nanodisks in therapy seems to be especially promising in the light of the of the fact that the highly tunable lipid environment could provide a milieu to the membrane-associated proteins which keeps them in solution without the use of detergents and maintain a structure which is more similar to the one they have when associated with biological membranes (Bayburt et al., 1998; Dominik and Kossiakoff, 2015). The reconstruction of a combination of antigens into membrane nanodisks has already been shown to generate protective immunity against viral challenge (Bhattacharya et al., 2010) and to raise anti-cancer immunity in mice (Kuai et al., 2017). Thus, the use of these membrane mimicking nanodisks to carry HSPs could provide a potential new formulation for HSP-based therapies.

## Conclusion

Immediately after an acute brain injury or infection, inflammation contributes to the efficient immune response, eliminating cellular debris and pathogens as a precursor to permitting tissue repair and regeneration. Similarly, in chronic neurodegenerative diseases, such as protein-misfolding disorders, immune cells can contribute to the elimination of toxic protein aggregates, at least in the initial phase. However, the long duration of the triggering stimuli results in excessive, chronic inflammation and uncontrolled macrophage activation finally contributing to further tissue injury and disease progression. Therefore, inflammatory processes should be tightly regulated to maintain the balance between remediation and the over-activated, harmful effects of the immune cells. Pharmacological manipulation of HSP expression, activity and localization might provide an opportunity to modulate beneficially the inflammatory processes in the brain. Thus, it seems rational to expect that the application of compounds with known HSP modulatory activity gradually will be tested for their applicability in the field of neuroinflammation. Alternatively, the delivery of HSP proteins to the disease-affected brain regions could also be expected to be exploited in future therapies. However, it is important to note that the full range of cell surface receptors functioning as HSP binding partners is not elucidated yet. Thus, aside from the technological implications of the delivery of the proteins, the proper surface receptor interactions and their physiological consequences should be appropriately evaluated for each brain area.

## Author Contributions

MT, BD, and BC, contributed to the conception and design in the manuscript. MT, BD, and BC wrote the manuscript. MT, BD, and BC reviewed the manuscript. MT, BD, and BC prepared the figures. All authors approved the final manuscript.

## Funding

This work was supported by funding from the National Research, Development and Innovation Office, Hungary (GINOP 2.3.2.-15.2016-00060, GINOP 2.3.2.-15.2016-00040 and GINOP 2.3.2.-15.2016-00001).

## Conflict of Interest Statement

The authors declare that the research was conducted in the absence of any commercial or financial relationships that could be construed as a potential conflict of interest.
